# Immune Cell Infiltration and Relevant Gene Signatures in the Tumor Microenvironment that Significantly Associates With the Prognosis of Patients With Breast Cancer

**DOI:** 10.3389/fmolb.2022.823911

**Published:** 2022-02-23

**Authors:** Qiang Xu, Xinghe Yan, Zhezhu Han, Xiuying Jin, Yongmin Jin, Honghua Sun, Junhua Liang, Songnan Zhang

**Affiliations:** Department of Oncology, Yanbian University Hospital, Yanji, China

**Keywords:** breast cancer, tumor microenvironment, gene, TME score, prognostic

## Abstract

Breast cancer is the most common malignancy and the leading cause of cancer-related deaths in women. Recent studies have investigated the prognostic value of the tumor microenvironment (TME)-related genes in breast cancer. The purpose of this research is to identify the immune-associated prognostic signature for breast cancer evaluate the probability of their prognostic value and compare the current staging system. In this study, we comprehensively evaluated the infiltration patterns of TME in 1,077 breast cancer patients downloaded from TCGA by applying the ssGSEA method to the transcriptome of these patients. Thus, generated two groups of immune cell infiltration. Based on two groups of low infiltration and high infiltration immune cell groups, 983 common differentially expressed genes were found using the limma algorithm. In addition, studying potential mechanisms, the GSEA method was used to indicate some pathways with remarkable enrichment in two clusters of immune cell infiltration. Finally, the seven immune-associated hub genes with survival as prognostic signatures were identified by using univariate Cox, survival, and LASSO analyses and constructed a TME score. The prognostic value of the TME score was self-validated in the TCGA cohort and further validated in an external independent set from METABRIC and GEO database by time-dependent survival receiver operation. Univariate and multivariate analyses of clinicopathological characteristics indicated that the TME score was an independent prognostic factor. In conclusion, the proposed TME score model should be considered as a prognostic factor, similar to the current TNM stage, and the seven immune-related genes can be a valuable potential biomarker for breast cancer.

## Introduction

Breast cancer is one of the major malignancies among females, and mortality remains the second main cause of cancer-related deaths worldwide (Siegel et al., 2019). Currently, similar to other cancers, the diagnosis of breast cancer mainly depends on pathological tests, imaging examinations, and the assessment of tumor markers (McDonald et al., 2016). The TNM staging system is a wildly used in clinical practice to guide treatment decisions and predict the prognosis of breast cancer patients. For better prognostic value, the current version of the TNM stage has been updated in the past decade, but it has not increased significantly (Nitsche et al., 2011). Recently, increasing attention has been paid to the research on the tumor microenvironment (TME) for its tremendous potential development capacity in the prognosis of patients with breast cancer (Baxevanis et al., 2021).

More studies have shown the role of the TME in cancer progression and therapeutic responses (Jiang et al., 2018). Emerging evidence related to TME revealed that it is crucial to patient outcomes. (Fridman et al., 2012). Successful elimination of tumors through immunotherapy requires activating the immune system. Unfortunately, the depletion or short-lived activation of immune cells and inhibition of microenvironment formation leads to resistance to immunotherapy (Tang et al., 2016). In addition to its effects on immunotherapy, the efficiency of chemotherapy and radiotherapy is also affected through its preexisting properties and induction therapy (Klemm and Joyce, 2015). Immune-associated genes and infiltration of immune cells in TME play a vital role in the properties of tumors, such as proliferation and development (Gonzalez et al., 2018). Therefore, characterizing the immune-associated genes with overall survival may present a prospective reference for breast cancer therapy and prognosis.

In this study, we used computational algorithms based on bulk tumor expression data that systematically profiled the immune cell landscape of the TME in 1,077 breast cancers (Aran et al., 2017). Then, we identified the signature of seven immune-associated hub genes that are related to the prognosis of differentially expressed genes (DEGs). Finally, we constructed a TME score that can be a novel prognostic factor for breast cancer instead of current the TNM stage.

## Materials and Methods

### Collection and Clustering of Breast Cancer Data

We systematically searched for the public breast cancer gene-expression datasets with fully annotated clinical data. We gathered 1,077 patient data on breast cancer genes and retrieve the corresponding clinical information from The Cancer Genome Atlas (TCGA) data portal (Weinstein et al., 2013). All data were analyzed using R (version 3.6.1) and R Bioconductor packages. The gene expression of GSE 103091 was obtained from Gene Expression Omnibus (GEO) database. 1904 samples from the METABRIC cohort were included in this study for further validation (Supplementary Table S1). We acquired 28 immune-related cells and types for further analysis (Charoentong et al., 2017). The different TME cell infiltration patterns with tumors were grouped using hierarchical agglomerative clustering (based on Euclidean distance and Ward’s linkage). The ssGSEA was performed on the types of immune infiltrating cells, immune-associated functions, and the pathways related to immunization in the expression data of breast cancer using the R package “GSVA”. A consensus clustering algorithm was applied to determine the number of clusters in the meta-data set and the Asian Cancer Research Group (ACRG) cohort to assess the stability of the discovered cluster. This procedure was performed using the Consensus Cluster Plus R package and was repeated 1,000 times to ensure the stability of the classification (Monti et al., 2003). According to the results of ssGSEA, low- and high- infiltration immune cell groups were classified in breast cancer samples from TCGA.

### Verification of the Effectuality of Immune Clustering

Using the R package “ESTIMATE”, gave immune and stromal cells in the TME scores based on the expression levels of specific genes (Yoshihara et al., 2013). ESTIMATE algorithm was used to count the tumor purity of 1,077 breast cancer samples to validate the effectuality of ssGSEA clustering and to create a heatmap and statistical map. Using the R package “ggpubr” generated the vioplots of ESTIMATE score, immune score, stromal score, and tumor purity in the two clusters. A principal component analysis (PCA) was applied using the R package “ggord” to further verify the cluster grouping. In order to investigate the difference among immune cell subtypes, we used hierarchical cluster by ConsensusClusterPackage to count the proportion of 28 immune cells in all breast cancer samples (Charoentong et al., 2017). We also used K-M analysis to validate the difference between two clusters by using the R package “survival”.

### Identification of Differentially Expressed Genes in Breast Cancer

The patients were grouped into two TME clusters based on immune cell infiltration for identifying the genes associated with TME cell infiltration patterns. The DEGs among these group was deterR package (Ritchie et al., 2015), which implements a Bayesian approach to estimate gene expression changes using moderated t-tests. DEGs among TME subtypes were determined by significance criteria (logFC>1 and *p* < 0.01) as implemented in the R package limma. Gene set enrichment analysis was performed between low- and high- immune cell infiltration clusters of DEGs using R package “ClusterProfiler” (Yu et al., 2012).

### Distinction and Conformation of Immune-Associated Gene Prognostic Signature for Breast Cancer

We downloaded clinical information with breast cancer in the TCGA dataset and used univariate Cox analysis to discern the immune-associated gene that was significantly associated with overall survival using the R “survival” package. Then, the LASSO regression analysis was performed to screen the hub genes according to the results in the univariate Cox regression analysis related to survival using the R “glmnet” package. A 1000-round cross-validation for tuning parameter selection was used to prevent overfitting and the partial likelihood deviance met the minimum criteria. Finally, we used the LASSO regression analysis to generated a prognostic signature of breast cancer via the expression level of immune-related hub genes and their relevant coefficients. The Kaplan-Meier (K-M) curves and time-dependent receiver-operating characteristic (ROC) were applied to assess the clinical prognostic capability of the TME score which was constructed by hub genes using the R packages “survival” and “survminer”. Moreover, to assess whether the TME score can indeed be rated as an independent factor of overall survival of breast cancer patients, multivariate and univariate Cox regression analyses were performed with TME score and clinicopathological characteristics as variables using the R package “survival” again.

### Statistical Analysis

The normality of the variables was tested using the Shapiro-Wilk normality test (Ghasemi and Zahediasl, 2012). For comparisons of two groups, statistical significance for normally distributed variables was estimated by unpaired Student’s t-tests, and non-normally distributed variables were analyzed using the Mann-Whitney *U* test. For comparisons of more than two groups, Kruskal-Wallis tests and one-way analysis of variance were used as nonparametric and parametric methods, respectively (Hazra and Gogtay, 2016). Correlation coefficients were computed using the Spearman and distance correlation analyses. Two-sided Fisher’s exact tests were used to analyze the contingency tests. To identify significant genes in the differential gene analysis we applied the Benjamini-Hochberg method to convert the *p* values to false discovery rates. The K-M method was used to generate survival curves for the subgroups in each data set, and the statistical significance of differences was determined using the log-rank test. The hazard ratios for univariate analysis were calculated using a univariate Cox proportional hazards model. A multivariate Cox regression model was generated using the heatmap function. All statistical analyses were conducted using the R software. Statistical significance was set at *p* < 0.05 (two-sided).

## Result

### Generation and Validation of Breast Cancer Clustering

**FIGURE 1:**
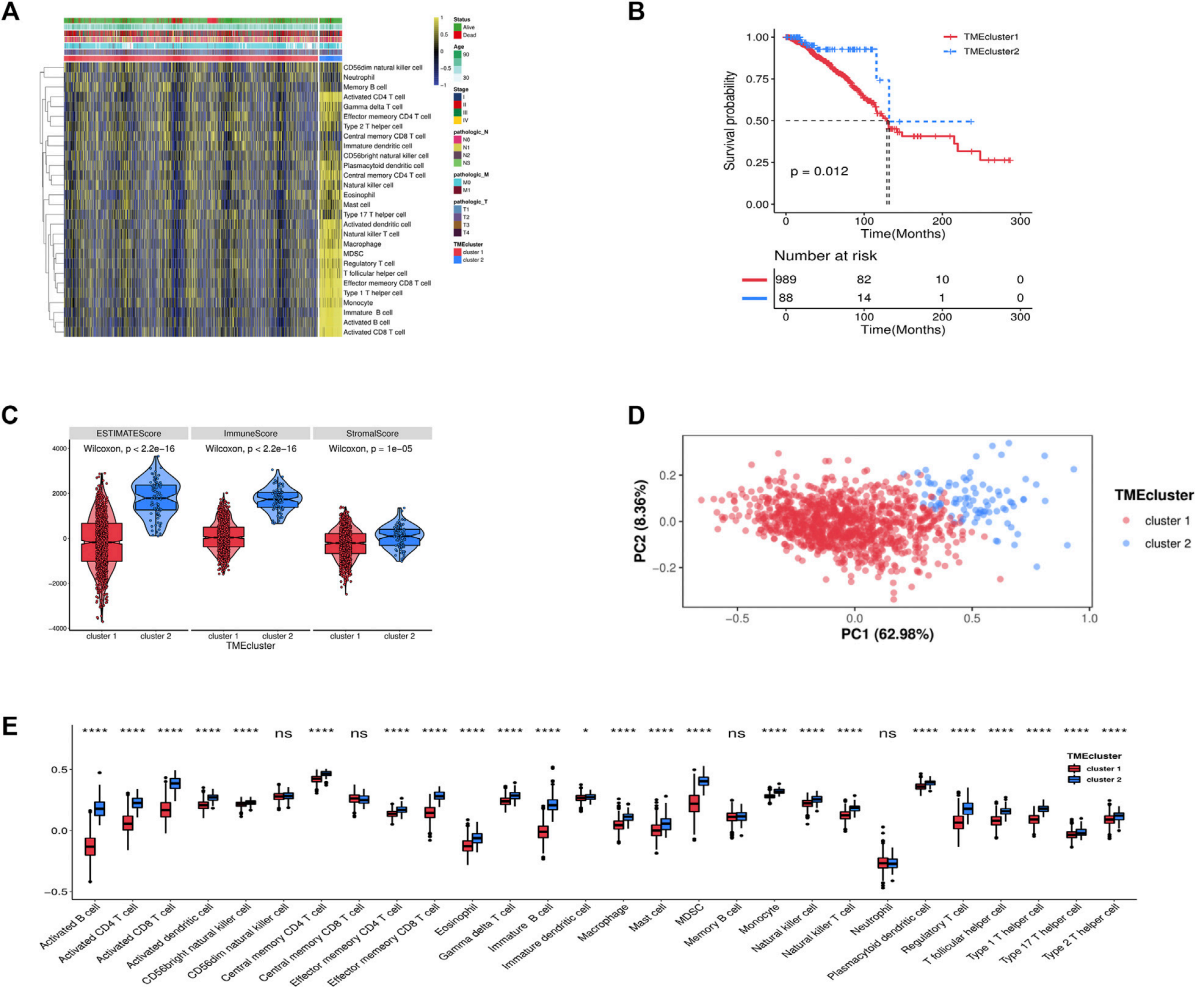
**(A)** Unsupervised clustering of TME cells for 1,077 patients in the TCGA cohort. **(B)** Kaplan-Meier curves for OS of 1,077 patients in TCGA cohort (log-rank test, *p* < 0.001). **(C)** Expression difference of ESTIMATE score, Immune Score, Stromal Score, and Tumor Purity in two clusters. **(D)** Principal component analysis (PCA) of two TME clusters modification pattern. **(E)** The fraction of TME cells in two clusters. The statistical difference of two TME clusters was compared through the Kruskal-Wallis test. *p < 0.05; **, p < 0.01; ***p < 0.001; ****p < 0.0001.

We obtained 1,077 breast cancer data from the TCGA. To realize the status of immune cell infiltration, the transcriptome data of breast cancer samples were grouped by the ssGSEA. A sufficient 28 immune-related cells and types in breast cancer samples were obtained. An unsupervised hierarchical clustering algorithm was used to assign the breast cancer samples into two clusters (cluster 1 and cluster 2) based on immune infiltration (Figure 1A). Also, we found that the low infiltration of immune cell cluster survival rate was significantly worse than the high infiltration cluster through the K-M curve in breast cancer patients (Figure 1B, Supplementary Table S2). The ESTIMATE score, stromal score, immune score, and tumor purity were calculated based on the infiltration level of breast cancer using the ESTIMATE algorithm to reflect the availability of the above clustering result. The violin plot has shown that the high infiltration of immune cell cluster (cluster 2) has a higher score than the low infiltration of immune cell cluster (cluster 1) in the stromal score, immune score, and ESTIMATE score (Figure 1C). The PCA plot further validated the precision of cluster grouping (Figure 1D). Figure 1E indicates that the significant differences in immune cell infiltration in the two TME clusters.

### Identification of DEGs Between Low and High Infiltration of Immune Cell Clusters

**FIGURE 2:**
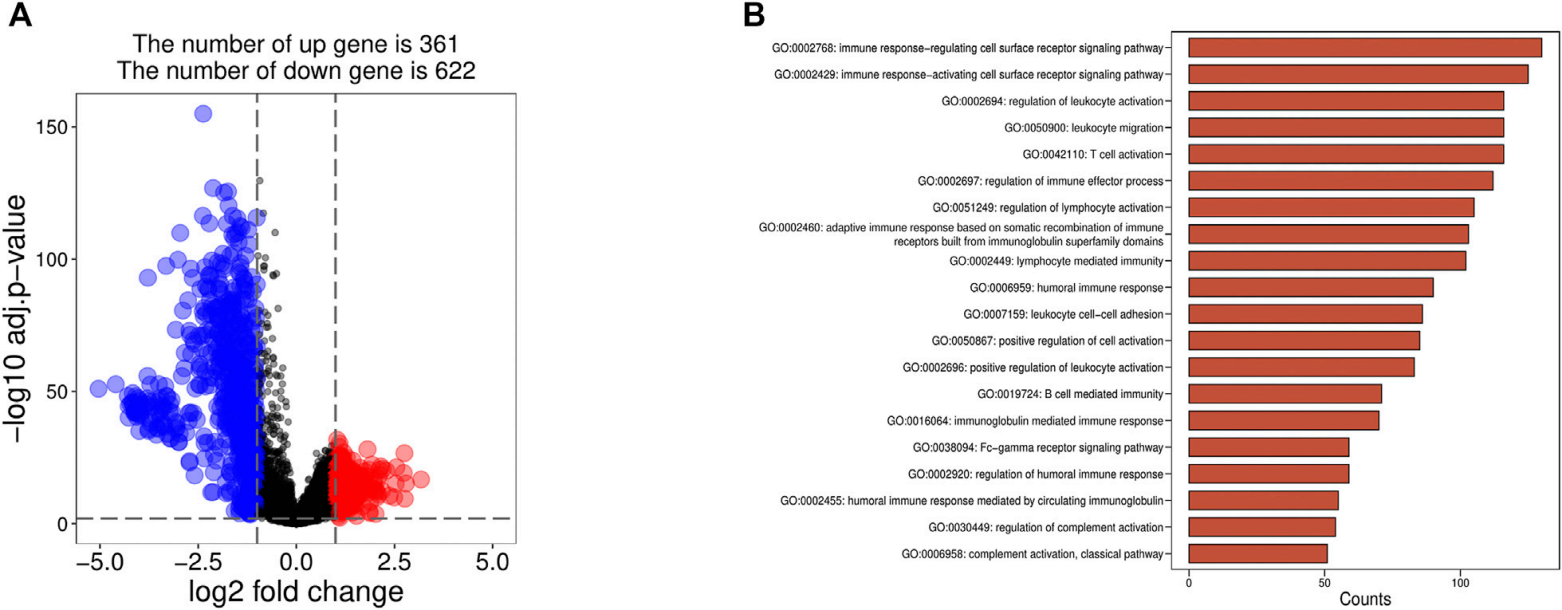
**(A) **The DEGs visualization was screened by limma. **(B)** GO enrichment analysis of the DEGs.

We identified the DEGs between the high infiltration of the immune cell group (cluster 2) and the low infiltration of the immune cell group (cluster 1) by using a significance criterion (logFC>1 and adj. *p* < 0.01). A total of 983 DEGs were preliminarily screened by limma and obtained 361 upregulated and 622 downregulated genes, respectively (Figure 2A).

### Functional Annotation by GSEA Enrichment Analysis

The GO analysis showed that genes in the high and low immune cell infiltration cluster in the TCGA database were almost related to immune response both in regulating and activating the cell surface receptor signaling pathway, regulation of leukocyte activation, and so on (Figure 2B).

### Distinction and Evaluation of Seven Immune-Associate Genes Prognostic Signature for Breast Cancer

**FIGURE 3:**
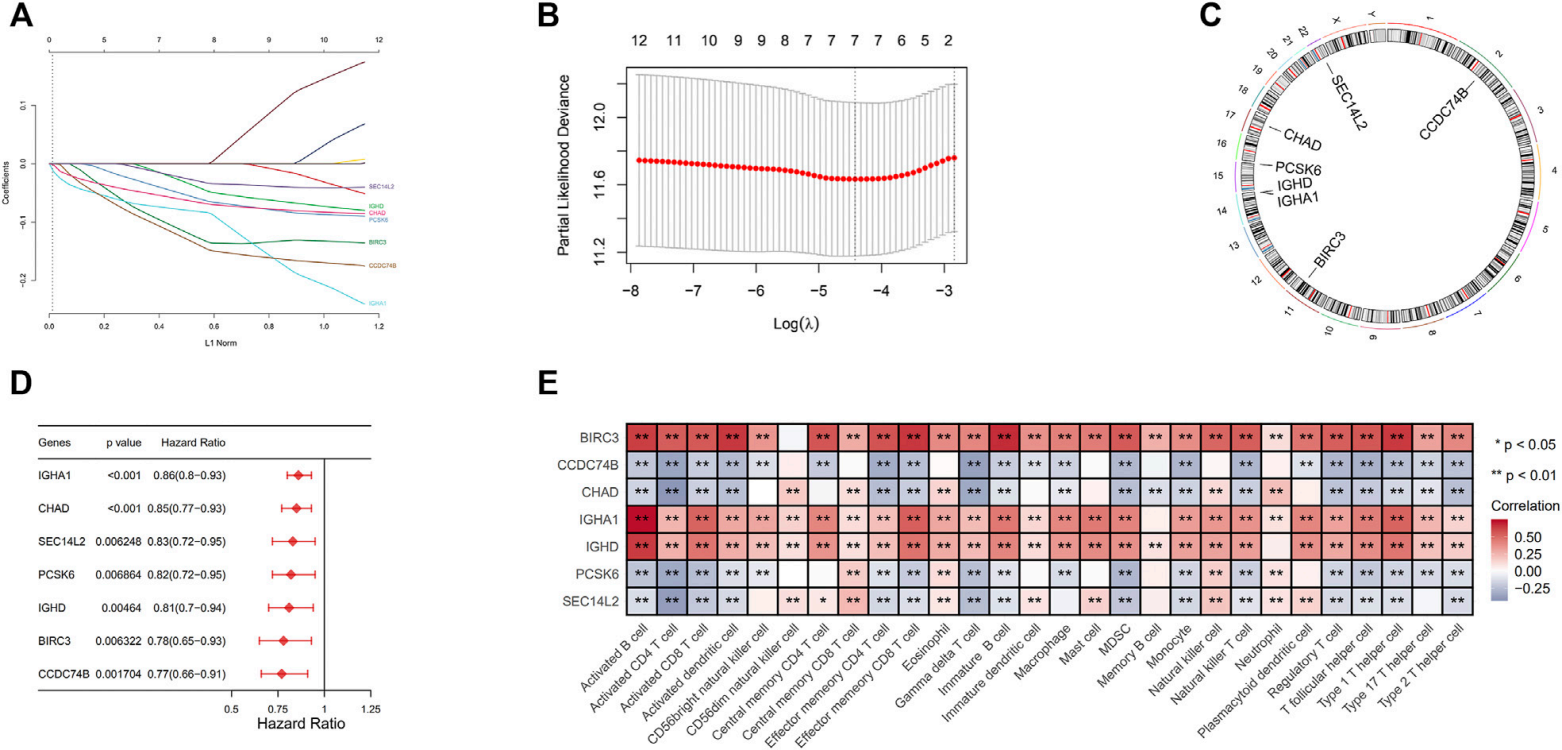
**(A)** The optimal penalty parameter values were confirmed by 1,000 round cross-validation. **(B)** Seven hub genes related to prognosis were analyzed by LASSO Cox analysis. **(C)** Each of seven genes on chromosomes was shown in Circos plots. **(D)** The contribution made by each of the seven genes to survival differences. **(E)** Correlation matrix of immune cell infiltration and the expression levels of seven immune-associated genes.

For further analysis, we used 1,077 breast cancer samples with complete clinical data. Univariate Cox regression analysis and LASSO regression analysis were used to identify seven immune-associated genes, including *SEC14L2, IGHD, IGHA1, CHAD, PCSK6, BIRC3*, and *CCDC74B*, which were most significantly associated with overall survival (Figures 3A,B). The details of immune-related genes are comprehensively displayed in the circus plot (Figure 3C). We further studied the prognosis value of each immune-related gene for breast cancer patients and demonstrated its correlation in immune cell infiltration (Figures 3D,E).

**FIGURE 4:**
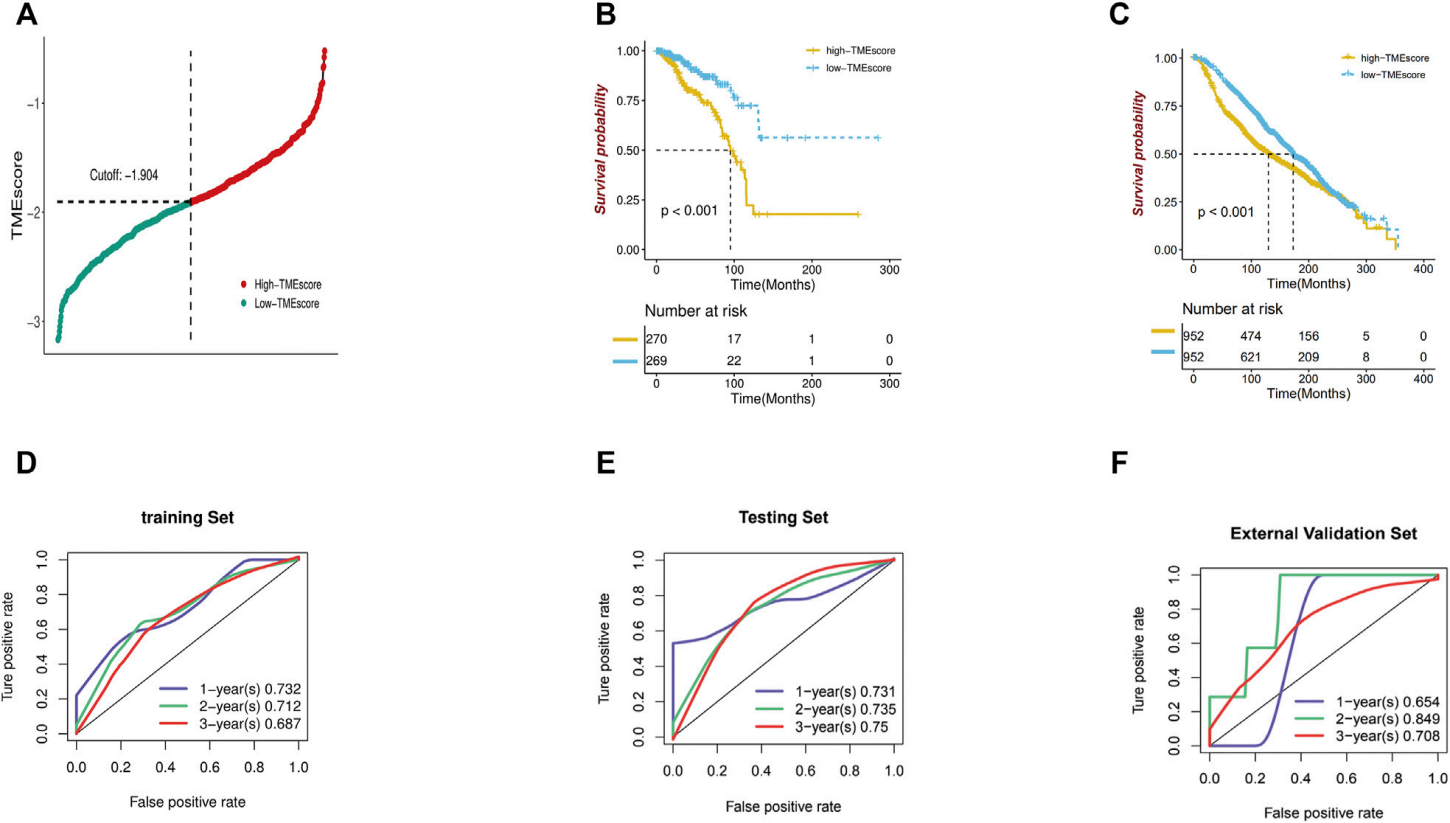
**(A–C)** The TME score and Kaplan-Meier curve analysis of seven immune-associated gene signature in TCGA-BRCA and METABRIC cohort. **(D–F)** ROC curves measuring the predictive values of the TME score at 1, 2, and 3 years in overall, training and external validation set, respectively.

Then, we assigned patients into high TME score and low TME score groups using the cutoff value (cut off -1.904) obtained with the Survminer package. Kaplan-Meier (K-M) curves and log-rank test were revealed that the low TME score group had a significantly better survival than the high TME score group (*p* < 0.001), showing that the TME score has an effective prognostic value (Figure 4A,B, Supplementary Table S2). Then we also test the prognosis value of seven immune-associated genes in METABRIC cohort. It showed that low TME score group also has better survival than high TME score group (Figure 4C).

### Assessment of Tumor Microenvironment Score as Independent Prognostic Factor in TCGA and GSE103091

The multivariate and univariate Cox regression analyses are applied to test whether the TME score calculated by the seven immune-associated hub genes was a latent independent prognostic factor. The Univariate and multivariate Cox regression analyses showed that TME score can be an independent prognostic factors (*p* < 0.001) (Table 1).

**TABLE 1:** Univariate and multivariate analyses of clinicopathological characteristics and TMEscore with overall survival in TCGA BRCA cohort.

Characteristics	Univariate analysis HR (95% CI)	*p*value	Multivariate analysis HR (95% CI)	Pvalue
TMEscore	2.875 (2.008–4.117)	<0.001	2.674 (1.76–4.061)	<0.001
Age	1.032 (1.02–1.045)	<0.001	1.029 (1.015–1.044)	<0.001
Stage				
Stage II	1.59 (0.92–2.749)	0.097	1.29 (0.525–3.166)	0.579
Stage III	3.033 (1.71–5.38)	<0.001	2.032 (0.578–7.144)	0.269
Stage IV	13.035 (6.426–26.438)	<0.001	5.306 (1.333–21.123)	0.018
pathologic_T				
T2	1.298 (0.863–1.953)	0.211	0.957 (0.497–1.842)	0.896
T3	1.576 (0.935–2.655)	0.087	0.895 (0.378–2.119)	0.801
T4	3.976 (2.142–7.378)	<0.001	1.283 (0.483–3.409)	0.617
pathologic_M	4.869 (2.906–8.157)	<0.001		
pathologic_N				
N1	1.851 (1.252–2.735)	0.002	1.42 (0.841–2.398)	0.19
N2	2.743 (1.641–4.585)	<0.001	1.812 (0.712–4.609)	0.212
N3	4.106 (2.27–7.428)	<0.001	1.778 (0.717–4.41)	0.214

HR, hazard ratio; CI, confidential interval.

In addition, the TME score stably maintained a powerful and independent factor in the training set, testing set, and external validation sets. Time-dependent ROC was applied to evaluate the precision of predicting overall survival of breast cancer at 1, 2 and 3 years between the high and low TME scores. The area under the ROC (AUC) values at 1,2,3 years were 0.732,0.712, and 0.687, respectively, in the training set and 0.731,0.735, and 0.75, respectively, in the internal testing. To validate whether our prognostic classifier had similar predictive ability in different populations, we applied it to the external validation set (GEO103091), and the result is 0.654, 0.849, and 0.708 at 1, 2, and 3 years, respectively (Figures 4D–F). The GO analysis of the low TME score group has showed that these were associated with B cell activation, cell chemotaxis, cell junction organization, cell matrix adhesion, cell substrate adhesion and more (Supplementary Figure S1). The KEGG analysis of the low TME score group has indicated that they were associated with the JAK-STAT signaling pathway, cytokine receptor interaction, leukocyte *trans*-endothelial migration, and so on (Supplementary Figure S2).

## Discussion

Breast cancer is the most frequent and deadly malignant tumor among women around the worldwide, because of its complicated TME, it is a highly heterogeneous disease (Sousa et al., 2019). The high heterogeneity of breast cancer exists in the molecular level of tumor cells, as well as in the TME (Baker et al., 2018). In addition, breast cancer tissue is not just composed of cancer cells, also mixed with several types of normal cells, such as immune cells, stromal cells, and fibroblasts (Bai et al., 2019). The relationship between TME and the properties of *BRCA*, such as tumor progression, invasion, and metastasis has been widely recognized (Bussard et al., 2016). Thus, we identified and validated the prognosis value of seven immune-associated genes with the survival of breast cancer datasets obtained from TCGA.

In this study, unsupervised hierarchical clustering algorithm was applied to classified the samples into two clusters based on the enrichment of 28 immune cell types. There were significant differences between low- and high- infiltration of immune cell clusters in the immune score, stromal score, ESTIMATE score, and tumor purity. In addition, the K-M analysis revealed that the breast cancer patients in the high immune cell infiltration cluster had a higher survival rate and that the survival rate of the two clusters was significantly different. We also discovered seven innovative immune-related genes based on the TCGA-BRCA cohort, which were successfully validated in an external independent set from METABRIC and GEO cohort. The overall survival time of the low-TME score group was significantly better than that of the high-TME score group. Univariate and multivariate Cox regression analyses showed that the seven immune-associated hub genes were an independent prognostic factors in both TCGA and GES 103091 datasets. Moreover, the AUC confirmed that the seven immune-related genes were comparable and superior to the TNM stage in predicting the overall survival of breast cancer patients. To further analyze the relationship between the TME score group and TNM stage. Therefore, these results suggest an excellent prediction capability for the seven immune-associated hub genes.

Seven immune-related hub genes, including *SEC14L2, IGHD, IGHA1, CHAD, PCSK6, BIRC3*, and *CCDC74B* were studied. Proprotein convertase subtilisin/Kexin type 6 (PCSK6) is a proteinase that regulates the proteolytic activity of various precursor proteins and protein maturation. A previous study revealed that PCSK6 significantly enhanced cell motility, migration, and invasion abilities when they overexpressed in MDA-MB-231 breast cancer cells *in vitro* (Lapierre et al., 2007). In addition, blocking PCSK6 expression in breast cancer MA-MB-231 cells inhibited their proliferation, invasion and migration abilities (Wang et al., 2015). In addition, PCSK6 was also reduced cell cycle arrest and prevented apoptosis of MDA-MB-231 cells and increased the expression level of the phosphorylated forms of ERK1/2 and WNT3A. Meanwhile, CCDC74B is a k-fiber crosslinker required for chromosomal alignment, thus by promoting the k-fiber stability and maintaining the spindle integrity to ensure proper chromosome alignment and cell division (Zhou et al., 2019). SEC14L2/TAP (tocopherol-associated protein) is a tocopherol-binding protein that regulates transcription and cholesterol metabolism (Porter, 2003). It is highly expressed in the breast, prostate, liver, and brain, the contrast was observed in many human tissues (Zimmer et al., 2000). A previous study demonstrated that TAP was downregulated in breast cancer; therefore, TAP/SEC14L2 may function as a tumor suppressor in breast tumors (Wang et al., 2009). BIRC3 (cellular IAP2) is a member of the human IAP family (Liang et al., 2020). It is overexpressed in malignant breast cancer compared to primary breast cancer, and is also linked to resistance to anti-cancer agents (Frazzi, 2021). Chondroadherin (CHAD) is a cartilage matrix protein thought to mediate adhesin in isolated chondrocytes (Camper et al., 1997). Low levels of CHAD have been associated with poor survival in hepatocellular carcinoma (Deng et al., 2017). IgA are produced in the airways and gastrointestinal tract, as well as in the lactating breast. IGHA1 and IGHA2 mRNA levels are highly correlated and are associated with improved prognosis with a higher immune activity in breast cancer (Larsson et al., 2020). IgD is a membrane-bound B cell receptor, and the B cell express IgD before the class switch. These results confirmed that the seven TME-associated immune genes affect the prognosis of patients with cancer.

We then further investigated the correlation between TME score and clinical characteristics in patients with breast cancer using the multivariate Cox and univariate Cox regression analyses. According to the result, the TME score showed good potential as an independent prognostic factor in patients with breast cancer (*p* < 0.001). Moreover, some studies have clarified the probability of TME score as a prognostic factor in several cancers. It is demonstrated its predictive value for immune checkpoint blockade (Zeng et al., 2019).

The limitations of our study are as followed. All the data and results were through a drying test and further research and confirmation is needed to reduce the bias from clinical practice, such as actual animal experiments.

In conclusion, we developed and validated the TME score which could be independent prognostic signatures for breast cancer based on the seven immune-related gene signatures. Our study may guide the prediction of prognosis and survival in patients with breast cancer and may provide potential targets for immunotherapy.

## Data Availability

The datasets presented in this study can be found in online repositories. The names of the repository/repositories and accession number(s) can be found in the article/Supplementary Material.
