# Supplementary material for: Immune Cell Infiltration and Relevant Gene Signatures in the Tumor Microenvironment that Significantly Associates With the Prognosis of Patients With Breast Cancer
Source: Front Mol Biosci. 2022 Feb 23;9:823911. doi: 10.3389/fmolb.2022.823911 (PMC8905140; doi:10.3389/fmolb.2022.823911)
Supplement: Supplementary file 1 [file DataSheet1.docx]

Supplementary Material

## Supplementary Figures


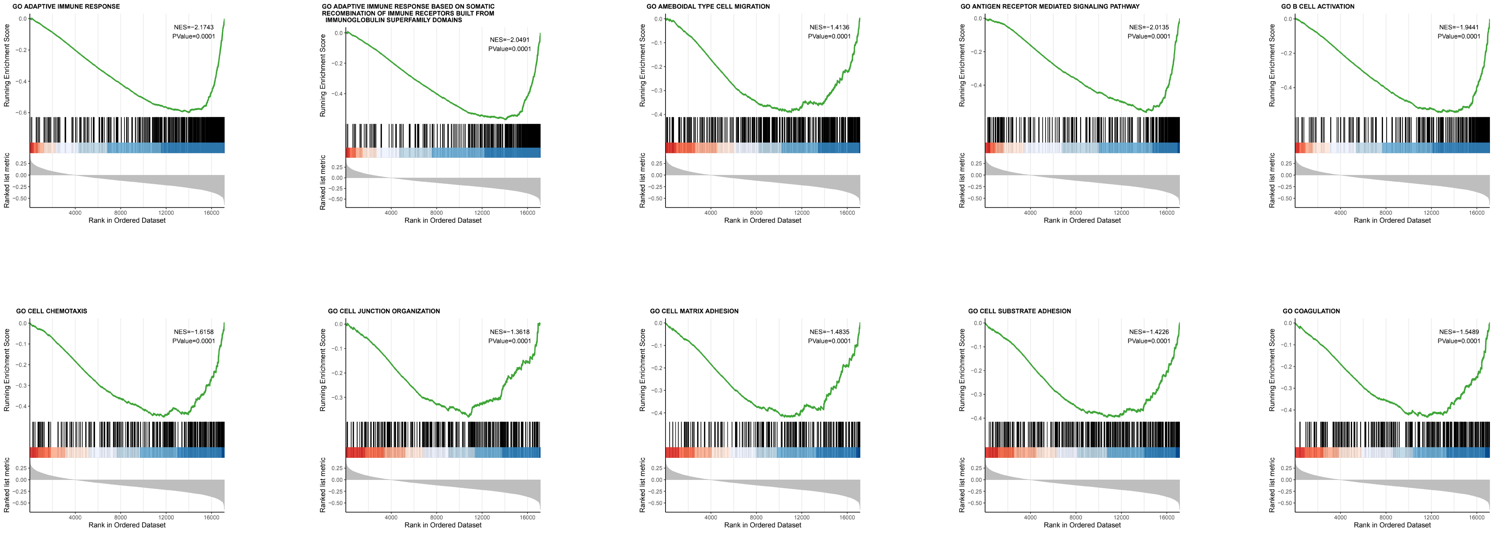


**Supplementary Figure 1.** GO enrichment analysis in low TME score group.

**
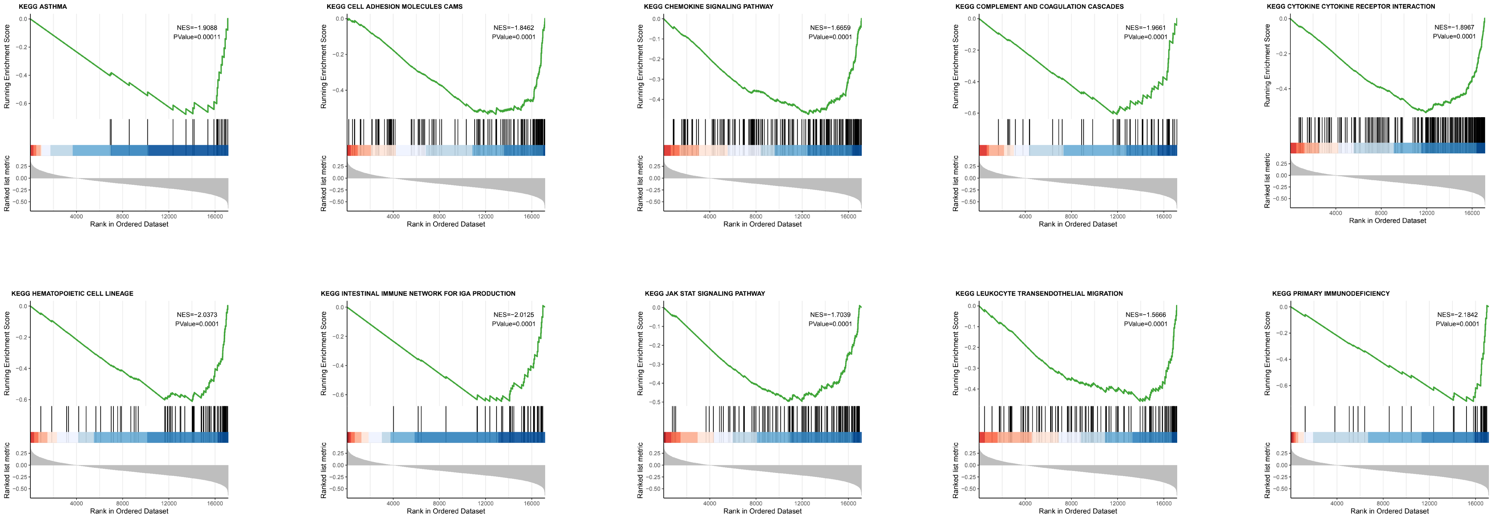
**

**Supplementary Figure 2.** KEGG pathways in low TME score group.
